# Association of the Calcification Score of the Abdominal Aorta, Common Iliac, and Renal Arteries with Outcomes in Living Kidney Donors

**DOI:** 10.3390/jcm12093339

**Published:** 2023-05-08

**Authors:** Luís Costa Ribeiro, Manuela Almeida, Jorge Malheiro, Filipa Silva, Diogo Nunes-Carneiro, La Salete Martins, Sofia Pedroso, Miguel Silva-Ramos

**Affiliations:** 1 School of Medicine and Biomedical Sciences, University of Porto, 4050-313 Porto, Portugal; 2 Nephrology and Kidney Transplantation Department, Centro Hospitalar e Universitário do Porto, 4050-366 Porto, Portugal; 3 Unit for Multidisciplinary Research in Biomedicine, School of Medicine and Biomedical Sciences, University of Porto, 4050-313 Porto, Portugal; 4 Urology Department, Centro Hospitalar e Universitário do Porto, 4050-366 Porto, Portugal

**Keywords:** kidney transplantation, vascular calcification, Agatston score, donor outcomes, expanded criteria donors

## Abstract

Background: Vascular calcification is an ever-more-common finding in protocoled pre-transplant imaging in living kidney donors. We intended to explore whether a connection could be found between the Agatston calcification score, prior to kidney donation, and post-donation renal function. Methods: This is a retrospective analysis of 156 living kidney donors who underwent living donor nephrectomy between January 2010 and December 2016. We quantified the total calcification score (TCaScore) by calculating the Agatston score for each vessel, abdominal aorta, common iliac, and renal arteries. Donors were placed into two different groups based on their TCaScore: <100 TCaScore group and ≥100 TCaScore group. The relationship between TCaScore, 1-year eGFR, proteinuria, and risk of 1 measurement of decreased renal function (eGFR < 60 mL/min/1.73 m^2^) over 5 years of follow-up was investigated. Results: The ≥100 TCaScore group consisted of 29 (19%) donors, with a median (interquartile range) calcification score of 164 (117–358). This group was significantly older, 56.7 ± 6.9 vs. 45.5 ± 10.6 (*p* < 0.001), had a higher average BMI (*p* < 0.019), and had a lower preoperative eGFR (*p* < 0.014). The 1-year eGFR was similarly diminished, 69.9 ± 15.7 vs. 76.3 ± 15.5 (*p* < 0.048), while also having an increased risk of decreased renal function during the follow-up, 22% vs. 48% (*p* < 0.007). Conclusions: Our study, through univariate analyses, found a relationship between a TCaScore > 100, lower 1-year eGFR, and decreased renal function in 5 years. However, a higher-than-expected vascular calcification should not be an excluding factor in donors, although they may require closer monitoring during follow-up.

## 1. Introduction

Studies estimate that, in highly developed nations, 1 in 10 people suffer from chronic kidney disease (CKD). Its diagnosis and treatment, especially in the more advanced stages of the disease, can severely impact the expectancy and quality of life [1]. When a patient reaches end-stage renal disease (ESRD), kidney transplantation has proved to be the best therapy, since the average life expectancy and quality of life of those who undergo kidney transplantation is higher than those who remain on dialysis [2,3]. After transplantation, estimates point to an increase of 10 years in overall life span, which varies between 3 and 17 years according to the patient group, with older patients having less benefit than younger recipients, although they still positively gain from the procedure [3,4,5].

Despite the increase in the number of kidney transplants performed annually, the waiting lists continue to grow, with the number of patients waiting for a kidney transplant increasing all around the globe. This increasing demand for grafts has led to the use of expanded criteria donors (ECD), which can be defined as any brain-dead donor aged > 60 years or a donor aged > 50 years with a history of hypertension, death resulting from a cerebrovascular accident, or terminal serum creatinine level ≥ 1.5 mg/dL [4,6]. These ECD kidneys have been demonstrated to have similar short- and long-term outcomes to standard criteria donors for the recipients and a clear decrease in mortality compared to patients who stayed on dialysis [5,6,7,8]. Parallelly, an expansion of selection criteria for living transplantation has started to occur, which has the potential to diminish further the number of end-stage renal patients on waiting lists [9]. Furthermore, with society becoming progressively older and obese and all the associated health problems, considering these donors is critical [10,11]. This expansion of selection criteria, even though living kidney donation has been performed for the better part of 8 decades, with minimal short-and long-term risk to the donor, might carry some increased peril to the donors, as their predicted medical futures are less precise than in the past. Thus, thoroughly evaluating these risk factors and comorbidities is essential to maintain donor safety [10,12].

Vascular calcification, a well-known consequence of atherosclerosis, is commonly associated with age, loss of kidney function, smoking, glucose resistance, dyslipidemia, hypertension, and mineral/bone parameter disorders [13,14]. Furthermore, over the past decade, coronary artery calcification has become a recognized objective predictor of cardiovascular events and other poor outcomes. Abdominal arterial calcification has also been associated with a higher incidence of cardiovascular disease, lower glomerular filtration rate (GFR), and all-round mortality in CKD patients and recipients of kidney grafts [15,16,17,18]. In donors, a seemingly healthy population, abdominal aortic calcification has been less researched. However, a study has claimed that aortic calcification might be an independent risk factor for pre-existing histopathological injuries in the allograft [19].

In order to evaluate and further advance the research into donors’ selection and risk-assessment parameters, this study aims to analyze the abdominal aortic, common iliac, and renal calcification in prenephrectomy living donors and its relationship with GFR, GFR trajectory, and proteinuria development, since ESKD, cardiovascular events, and mortality after donation are such rare outcomes for a 5-year follow-up study. The quantification of calcification in the arteries is calculated through the Agatston score, which is based on the size of lesions positively weighted by a categorical factor of the calcium density on abdominal and pelvic CT imaging taken prior to kidney transplantation, and which is primarily performed for surgical planning. As imaging data are available, non-contrast-enhanced CT-based quantification of aortic, iliac, and renal calcification can be used for risk stratification of patients screened for kidney transplantation without the need for additional procedures [20,21].

## 2. Materials and Methods

### 2.1. Subjects and Study Design

This is a retrospective study of a single transplant center, reviewing the clinical records of living kidney donors, with nephrectomies performed at Centro Hospitalar Universitário do Porto (CHUP) between January 2010 and December 2016. This study was approved by the Ethics Committee and Department for Education, Training, and Research of CHUP/ICBAS, as the data for the study were retrieved from patients’ electronic and paper medical records, while always maintaining their anonymity. Of the 169 donor nephrectomies performed in this 6-year timeframe, 9 patients were excluded as their CT scans were unavailable for examination and another 4 patients were additionally excluded due to not having an evaluation of preoperative eGFR or eGFR at 1 year. Thus, the remaining 156 donors defined our study cohort.

Donor characteristics regarding demographic information, anthropometric measurements, prior or current diagnosis or treatment for hypertension, diabetes mellitus and dyslipidemia, laboratory data, and imaging before nephrectomy were recorded, as well as during the 5-year follow-up period. The CT scans, performed according to the pre-transplant screening protocol, with 1 of 2 multidetector-row CT scans available at our institution (a 64-detector GE VCT LightSpeed^®^ (Chicago, IL, USA, or a 16-detector GE Brightspeed^®^ (Chicago, IL, USA), were examined using the 3D Slicer^®^ software, version 4.11, which allows for the calculation of the calcification score of the abdominal aorta, from the branch of the celiac trunk to the branch of the common iliac artery, and of the common iliac and renal arteries, along their entire length. The calcification scores were calculated through the Agatston method, using a 5 mm CT slice thickness and detection threshold ≥ 130 Hounsfield units involving ≥1 mm^2^ area or 3 adjacent pixels. The individual Agatston scores were calculated by multiplying each area of interest by a weighted score assigned to the highest density of calcification (1 for 130–199 HU, 2 for 200–299 HU, 3 for 300–399 HU, and 4 for >400 HU) within the individual area. The total calcification score (TCaScore) was the cumulative sum of the Agatston scores of all calculated areas and vessels (CaScore) [20,21].

### 2.2. Outcome Parameters

The parameter of interest was the total calcification score (TCaScore) at donation. Donors were stratified into two groups by their baseline TCaScore, either absent-to-minimal calcification, <100 TCaScore, or moderate-to-severe calcification, ≥100 TCaScore. These two categories were defined considering our small population and in accordance with larger studies on vascular calcification, particularly the MESA study. The glomerular filtration rate (eGFR) was estimated employing the 2021 Chronic Kidney Disease Epidemiology Collaboration equation (CKD-EPI). A post-donation eGFR < 60 mL/min per 1.73 m^2^ was considered to be decreased and was evaluated yearly during follow-up, in addition to being used as a main endpoint. Regarding other relevant medical conditions, post-donation diabetes mellitus was defined as fasting glycemia ≥ 126 mg/dL or the use of insulin and/or other hypoglycemic agents. During visits, hypertension was classified as a prescription of antihypertensive medications or a registered blood pressure ≥ 140/90 mmHg. Dyslipidemia, before or after nephrectomy, was considered present when donors either used hypolipidemic agents, had total cholesterol > 200 mg/ dL, LDL > 130 mg/dL, triglycerides > 150 mg/dL, or HDL < 40 mg/dL. On urinary analysis, proteinuria was defined by the presence of urine random protein > 15 mg/dL or 24 h protein > 300 mg/day.

### 2.3. Statistical Analysis

Continuous variables are reported as mean + standard deviation (SD) or median (interquartile range (IQR)) and categorical variables are presented as frequency and percentage. The categorical data included were compared using the Pearson chi-square test or Fisher exact test and continuous variables were compared with the Student’s *t*-test or Mann–Whitney U-test, as appropriate. Spearman’s correlation test assessed correlations between eGFR values, at donation and at 1-year follow-up, and total calcification scores (TCaScore).

Linear prediction of eGFR at 1-year follow-up was analyzed through a univariate and multivariable linear regression model, and risk factors for an eGFR < 60 mL/min per 1.73 m^2^ at 1 year were calculated through a univariate multivariable logistic regression. To establish comparisons between covariates regarding the strength of association, the increment in continuous variables was assessed per standard deviation.

Donor eGFR slope between 1 and 5 years after transplant was assessed by a univariate and multivariable linear mixed regression model that imputed subject-specific random effects (intercept and slope defined as eGFR at 1 year and time in years, respectively) on an unstructured covariance matrix. The dependent variable was all eGFR measurements and the independent variables were entered as 2-way interaction terms between them and the time (in years) variable. All 156 donors were studied and a median of 3 (IQR: 2–4) annual measurements of eGFR were available. All multivariable models were constructed by including variables with a univariate *p*-value < 0.150.

Risk predictors of the first measurement with an eGFR < 60 mL/min/1.73 m^2^ or first proteinuria (as defined above) were analyzed using univariate and multivariate Cox regression models. Previous outcomes-free survival curves, between donors with TCaScore < 100 and TCaScore ≥ 100, were depicted by Kaplan–Meier curves and comparisons were made by the log-rank test.

Statistical analysis was performed using STATA/MP^®^, version 15.1 (Stata Corp, College Station, TX, USA). A 2-sided *p*-value < 0.05 was considered statistically significant.

## 3. Results

### 3.1. Overall

Of the 156 donors present in this cohort, 29 (19%) presented with a total calcification score (TCaScore) ≥ 100 based on their pre-transplant CT scans. The majority of donors were female (71%), presented with an average body mass index (BMI) at donation of 25.1 ± 3.5 kg/m^2^, with body mass index (BMI) between groups being 24.8 ± 3.5 kg/m^2^ vs. 26.4 ± 2.9 kg/m^2^ in the TCaScore < 100 and TCaScore ≥ 100 groups, respectively (*p* < 0.019). Overall, the average donor age was 47.6 ± 10.9, the mean preoperative glomerular filtration rate (GFR) was 105.3 ± 13.6 mL/min/1.73 m^2^, and there were only 20 active smokers at the time of donation.

### 3.2. Main Differences between Groups

The median (interquartile range) total calcification score was 0 (0–50), including the TCaScore ≥100 group with median (interquartile range) calcification of 164 (117–358). There were notable differences between the TCaScore groups regarding age; the TCaScore < 100 group presented with 45.5 ± 10.6, contrasting with the mean age of 56.7 ± 6.9 in the TCaScore ≥ 100 group (*p* < 0.001). Dyslipidemia was more commonly found in TCaScore ≥ 100 donors, with 21 (75%; *p* < 0.027), along with a higher likelihood of hypertension history, with 26 donors (17%) overall, 10 (34%) of whom were in the TCaScore ≥ 100 group (*p* < 0.004). The TCaScore ≥ 100 group had lower preoperative eGFR values, 106.6 ± 13.4 mL/min/1.73 m^2^ vs. 99.7 ± 13.5 mL/min/1.73 m^2^ (*p* < 0.014) (Table 1).

#### 3.2.1. At 1 Year

At the first year of postoperative follow-up, the mean eGFR was 75.1 ± 15.7 mL/min/1.73 m^2^, although when analyzing each group separately, the TCaScore ≥ 100 group more frequently had delayed renal function recovery, 69.9 ± 15.7 mL/min/1.73 m^2^ vs. 76.3 ± 15.5 mL/min/1.73 m^2^ (*p* < 0.048). The same group of donors also showed an increase in the probability of post-donation eGFR < 60 mL/min/1.73 m^2^ at 1-year follow-up, with 8 donors (28%) vs. 16 donors (13%; *p* < 0.044) (Table 2). Linear and logistic regression analysis for eGFR and eGFR < 60 mL/min/1.73 m^2^, respectively, showed a univariate statistical significance for both (*p* < 0.048; *p* < 0.049), although the same cannot be said when a multivariable analysis, adjusted for age, sex, hypertension, BMI, and eGFR at donation, was performed (*p* < 0.887; *p* < 0.651).

#### 3.2.2. At 5 Years

**Figure 1:**
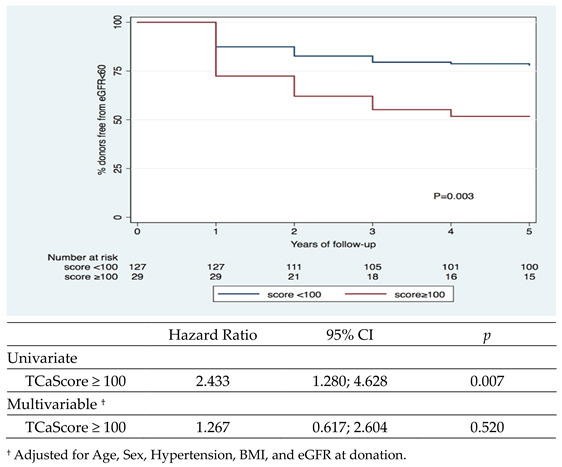
Risk of 1 measurement of decreased eGFR, <60 mL/min/1.73 m^2^, over the course of the 5-year follow-up in donors with and without moderate-to-severe abdominal aortic, iliac, and renal calcification.

The risk of 1 measurement of decreased eGFR, <60 mL/min/1.73 m^2^, over the course of the 5-year follow-up was 27% for the entire donor pool, along with 22% for the TCaScore < 100 group and 48% for the TCaScore ≥ 100 group (Univariate = *p* < 0.007; Figure 1). Moreover, the chance of proteinuria in the same period was 33% (31% vs. 38%; Univariate = *p* < 0.433; Multivariate (adjusted for age, sex, hypertension, BMI, eGFR at donation) = *p* < 0.136).

**Figure 2:**
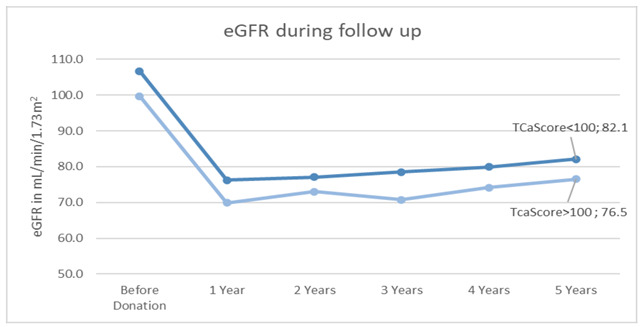
Kidney function variation over the 5-year follow-up in donors with and without moderate-to-severe abdominal aortic, iliac, and renal calcification.

During the entire follow-up, the group with an elevated TCaScore always averaged a lower eGFR (Figure 2). However, significant outcomes over the years were always parallelly associated with lower eGFR at donation and traditional risk factors such as age, hypertension, and dyslipidemia, as previously mentioned. The estimated eGFR slope from 1 to 5 years of post-donation follow-up was assessed by a univariate and multivariable linear mixed regression model but wielded no statistically significant results.

## 4. Discussion

In this retrospective single-center study, we analyzed the clinical implication of vascular calcification score, through the Agatston method, and its association with renal function. Regarding the presence of moderate-to-severe calcification (TCaScore ≥ 100), compared with minimal to absent calcification of their aorta, iliac, and renal arteries (TCaScore < 100), we found that higher degrees of vascular calcification were observed significantly more often in older patients, those with higher BMI, hypertension, dyslipidemia, and lower eGFR at donation, corroborating the known relationship between these traditional risk factors and arterial calcification [13,14,15,16].

At 1-year follow-up, donors with TCaScore ≥ 100 exhibited a trend of lower mean eGFR and were at a significant risk for decreased renal function (eGFR < 60 mL/min/1.73 m^2^); nevertheless, this trend was parallelly associated with more advanced age and other comorbidities in donors. Thus, after correcting for age difference, sex, the presence of hypertension, and BMI, all known risk factors for lower eGFR at donation, there was no difference in predicting eGFR at 1 year or risk eGFR < 60 mL/min/1.73 m^2^ between the 2 groups. Likewise, the absolute loss of the average kidney function between both groups was similar, with the higher calcification group losing 28.8 mL/min/1.73 m^2^ and the minimal calcification group dipping 30.3 mL/min/1.73 m^2^.

Several previous studies have evaluated aortic calcification, but relatively few have focused on donors and their outcomes. The only one, to our knowledge, is the study of 287 donors by Yoon et al., which arrived at similar findings to ours: a calcification score of the abdominal aorta valve valued over 100 raised the probability of GFR < 60 mL/min per 1.73 m^2^, at 6 months of follow-up, and was associated with histologic findings of nephrosclerosis [22]. Studies featuring abdominal arteries’ calcification and graft outcomes are more common, with clear similarities and parallels with our findings. Ichii et al. arrived at the statistically independent association between lower eGFR and the quantitative degree of aortic calcification in non-dialysis patients [17]. Moreover, in 2021, Benjamens et al. demonstrated that pre-transplant aorto-iliac calcification is associated with 1-year eGFR in univariate linear regression analyses [23]. Tatami et al. identified trends between abdominal aortic calcifications and cardiovascular events in asymptomatic non-dialysis patients (median eGFR, 43.2 + 17.7 mL/min/1.73 m^2^) [24].

We further evaluated the effects of TCaScore over a longer period and identified a non-causal inverse association between the 2 calcification groups and the risk of 1 measurement of decreased eGFR, <60 mL/min/1.73 m^2^, over the course of the 5-year follow-up, in univariate analysis. Donors lose approximately 30% of their pre-donation glomerular filtration rate after they go through compensatory hypertrophy and hyperfiltration. Donor nephrectomy itself does not appear to cause long-term loss of eGFR at a higher rate than that seen in the general population, which can be seen in Figure 2, as both groups regain function over the course of the years [25,26,27]. Although certain pre-donation characteristics, for instance, arterial calcification and lower pre-donation eGFR, both present in the TCaScore >100 group, and even remaining kidney volume indexed to weight, might put donors at risk of decreased compensatory mechanisms, thus increasing the risk of having a measure of eGFR < 60 mL/min/1.73 m^2^, and even future post-donation kidney injury [26,28].

The screening protocol for renal donors is thorough for any cardiac or kidney disease, diabetes, or other comorbidities that might render the transplant a higher-than-accepted risk for the donor [10]. CT scans are also routinely performed in order to evaluate the number of renal vessels, kidney volume, and the presence of renal artery atheroma, which has been associated with graft thrombosis and even mortality [29]. It stands to reason that using the same CT data to further evaluate the calcification score of the aorta, iliac, and renal arteries will only optimize the donor’s pre-donation risk assessment, especially in expanded criteria living donors, gaining further safety and information for an even more common donor profile.

This study has a number of important limitations. Firstly, the retrospective observational nature of the study design, of which the selection of donors depended on the availability of a pre-transplant CT scan, makes this study prone to selection bias and confounding, even though we controlled for important demographic factors, comorbidities, and baseline eGFR. Secondly, the quantification technique for vascular calcification in this study was performed by an isolated examiner. However, the Agatston method is considered reliable and reproducible with reasonable interscanner variability [30]. Thirdly, the relatively small study cohort of 156 and follow-up period of 5 years for nearly every donor, which despite being, to our knowledge, the longest of any CT-based studies on vascular calcification, is lacking compared to larger registry studies, which are required to comprehend the conclusive implications of vascular calcification score in kidney donors’ outcomes.

## 5. Conclusions

In conclusion, this study proved that pre-transplant vascular calcification score is correlated with a 1-year eGFR and a risk of decreased renal function in 5 years in univariate, but not in multivariable linear regression, analyses. Increased donor age and lower preoperative eGFR were significant confounders for this association, not to mention sex, hypertension, and BMI. These results underline that living donors from expanded criteria living donors are safely stratified with current screening practices, despite the increase of older and overweight donors with comorbidities. Despite this, based on these findings, living donors with severe calcification on CT scans may require close monitoring and follow-up.

## Figures and Tables

**Table 1:** Baseline characteristics and calcification scores.

	Total N = 156	TCaScore < 100 N = 127 (81%) *	TCaScore ≥ 100 N = 29 (19%) *	*p*
Age at donation, mean ± SD *	47.6 ± 10.9	45.5 ± 10.6	56.7 ± 6.9	<0.001
Female donor, n (%)	111 (71)	94 (74)	17 (59)	0.099
BMI at donation, mean ± SD *	25.1 ± 3.5	24.8 ± 3.5	26.4 ± 2.9	0.019
Hypertension at donation, n (%)	26 (17)	16 (13)	10 (34)	0.004
Smoker, n (%)	20 (13)	16 (13)	4 (14)	0.768
Dyslipidemia at donation, n (%) Missing = 3	86 (56)	65 (52)	21 (75)	0.027
eGFR at donation, mean ± SD *	105.3 ± 13.6	106.6 ± 13.4	99.7 ± 13.5	0.014
TCaScore, median (P25–P75) [P10–P90] *	0 (0–50) [0–262]	0 (0–0) [0–35]	269 (182–564) [125–1276]	<0.001
CaScore Abdominal Aorta, median (P25–P75) [P10–P90] *	0 (0–10) [0–151]	0 (0–0) [0–12]	164 (117–358) [5–815]	<0.001
CaScore Renal Artery, median (P25–P75) [P10–P90] *	0 (0–0) [0–0]	0 (0–0) [0–0]	0 (0–0) [0–7]	<0.001
CaScore Common Iliac Artery, median (P25–P75) [P10–P90] *	0 (0–0) [0–84]	0 (0–0) [0–9]	99 (18–247) [0–610]	<0.001

* SD, standard deviation; BMI, body mass index, expressed in kg/m^2^; eGFR, estimated glomerular filtration rate, expressed in mL/min/1.73 m^2^; TCaScore, Total Calcification Score; CaScore, Calcification Score.

**Table 2:** Outcomes’ 1-year post-donation follow-up.

	Total N = 156	TCaScore < 100 N = 127 (81%) *	TCaScore ≥ 100 N = 29 (19%) *	*p*
eGFR, mean ± SD *	75.1 ± 15.7	76.3 ± 15.5	69.9 ± 15.7	0.048
eGFR <60, n (%) *	24 (15)	16 (13)	8 (28)	0.044
Proteinuria, n (%) Missing = 25	24 (18)	17 (17)	7 (25)	0.303
Dyslipidemia, n (%) Missing = 12	89 (62)	69 (59)	20 (71)	0.243

* SD, standard deviation; eGFR, estimated glomerular filtration rate, expressed in mL/min/1.73 m^2^; TCaScore, Total Calcification Score.

## Data Availability

Data are unavailable due to privacy and ethical restrictions.
